# Circ_0002669 promotes osteosarcoma tumorigenesis through directly binding to MYCBP and sponging miR-889-3p

**DOI:** 10.1186/s13062-024-00466-1

**Published:** 2024-04-03

**Authors:** Ying Zhang, Yizhou Zhan, Zhaoyong Liu, Huancheng Guo, Dongchen Liu, Chuangzhen Chen

**Affiliations:** 1 Department of Radiotherapy, Cancer Hospital of Shantou University Medical College, No. 7 Raoping Road, 515041 Shantou, Guangdong PR China; 2 Department of Clinical Research Center, Cancer Hospital of Shantou University Medical College, No. 7 Raoping Road, 515041 Shantou, Guangdong China; 3 Department of Orthopaedics, First Affiliated Hospital of Shantou University Medical College, No.57 Changping Road, 515041 Shantou, Guangdong China

**Keywords:** Osteosarcoma, Circ_0002669, MYCBP, miR-889-3p

## Abstract

Circular RNAs (circRNAs) are a class of highly multifunctional single-stranded RNAs that play crucial roles in cancer progression, including osteosarcoma (OS). Circ_0002669, generated from the dedicator of cytokinesis (DOCK) gene, was highly expressed in OS tissues, and negatively correlated with OS patient survival. Elevated circ_0002669 promoted OS cell growth and invasion in vivo and in vitro. By biotin pulldown and mass spectroscopy, we found that circ_0002669 directly bound to MYCBP, a positive regulator of c-myc, to prevent MYCBP from ubiquitin-mediated proteasome degradation. In addition, circ_0002669 interacted with miR-889-3p and served as a miRNA sponge to increase the expression of MYCBP, as determined by luciferase assays and RNA immunoprecipitation. Functional rescue experiments indicated MYCBP acted as a key factor for circ_0002669- and miR-889-3p-regulated OS cell proliferation and migration. Increased expression of c-myc-associated genes, such as CCND1, c-Jun and CDK4, were found in circ_0002669- and MYCBP-overexpressing OS cells. Our data thus provide evidence that circ_0002669 promotes OS malignancy by protecting MYCBP from protein ubiquitination and degradation and blocking miR-889-3p-mediated inhibition of MYCBP expression.

**Supplementary Information:**

The online version contains supplementary material available at 10.1186/s13062-024-00466-1.

## Introduction

Osteosarcoma (OS) is one of the most common malignant bone tumors that often occurs in adolescents, and has a 5-year survival rate of less than 20% after diagnosis of metastases [1]. Increasing evidence shows the crucial roles of genetic and epigenetic dysregulation in the generation and development of OS [2]. CircRNA plays important and diverse roles in regulating cell differentiation, pathogenesis of multiple diseases, and metastasis and stemness of tumor cells [3]. The function and expression of circRNA in OS has been demonstrated in several studies [4]. For example, circ_0001422 is upregulated in OS tissues, compared with matched noncancerous tissues, and correlates with poor survival [5]. However, the function and molecular mechanism of circRNA still need to be explored.

Mechanistically, circRNA acts as a competing endogenous RNA (ceRNA) by decoying miRNAs to regulate the expression of downstream genes [6]. Inhibition of circFAT1 effectively prevents the migration, invasion, and tumorigenesis of OS cells in vitro and in vivo by sponging miR-375 to upregulate the expression of YAP1 [7]. CircTADA2A acts as a tumor promoter in OS to increase cell migration, invasion and proliferation via the miR-203a-3p/CREB3 axis [8]. In addition, circRNAs can directly bind to protein and manage the transcription of downstream genes. For example, circECE1 interacts with c-Myc to prevent c-Myc ubiquitination and degradation, thus mediating OS energy metabolism [9].

Dedicator of cytokinesis (DOCK)1, a guanine nucleotide exchange factor (GEF) family member, has been reported to be not only involved in the initiation of cancer, but also the modulation of malignant phenotypes in glioma, liver cancer and breast cancer [10, 11]. Several circRNAs generated from DOCK1 have been reported to be tumor promoters. For example, circDOCK1 (hsa_circ_0020397) interference suppresses colorectal cancer cell growth and metastasis, and increases apoptosis via miR-132-3p/USP11 [12]. In thyroid cancer, circDOCK1 (hsa_circ_1007211) contributes to thyroid carcinogenesis through inhibition of the miR-124/JAK/STAT/AMPK signaling pathway [13]. Here, we identify a circRNA, hsa_circ_0002669 that is generated from the DOCK1 gene, and explore its expression and function in OS.

Previous studies demonstrated that c-MYC binding protein (MYCBP), an 11-kDa protein, promotes tumorigenesis by binding to c-MYC, an oncogenic protein, and stimulates the transcriptional activation of c-MYC and expression of MYC-dependent downstream genes. C-MYC-activated genes subsequently mediate several tumor malignant properties, including cell proliferation, invasiveness and metastasis [14]. Recently published studies show that MYCBP plays a tumor promoter role in glioma, hepatocellular carcinoma and esophageal squamous cancer [15, 16]. Moreover, MYCBP is the target gene for several miRNAs and enrolled in tumor progression through binding miRNAs, such as miR-495-3p, miR-574-5p and miR-26b-5p [17, 18]. However, it still remains unknown whether MYCBP plays a role in OS.

In this paper, we found circ_0002669 is highly expressed in OS tissues and correlates with poor survival of OS patients. We further explored the function of circ_0002669 and found it elevates the expression of MYCBP via protecting MYCBP from proteasome degradation and inhibition by miR-889-3p, resulting in OS cell proliferation and invasion. Our results indicate that circ_0002669 functions as a tumor promoter and can be considered a therapeutic target for OS.

## Materials and methods

### Patient samples and cell culture

Fresh OS tissues and corresponding normal tissues were obtained from the First Affiliated Hospital of Shantou University Medical College and Sun Yat-sen University Cancer Centre. Formalin-fixed and paraffin-embedded OS (*n* = 72) and normal tissue (*n* = 6) slides as well as fresh OS tissues (*n* = 12) and normal tissues (*n* = 5) were obtained from biopsies of OS patients. All samples were obtained before chemotherapy or radiotherapy. Written informed consent was obtained from each patient before entering this study, and all study protocols were approved by the Ethics Committee of the Cancer Hospital of Shantou University Medical College (NO. 2,022,047).

Human embryonic kidney (HEK) 293T (RRID: CVCL_0063), osteoblast hFOB1.19 (RRID: CVCL_3708) and OS cell lines MG63 (RRID: CVCL_0426), U2OS (RRID: CVCL_0042), HOS (RRID:CVCL_0312)and Saos2(RRID:CVCL_0548),were used in this study. MG63, HOS, Saos2 and U2OS cells were cultured in Dulbecco’s modified Eagle’s medium (DMEM, Gibco, NY, USA) supplemented with 1% penicillin-streptomycin and 10% fetal bovine serum (FBS, Gibco), and maintained at 37 °C in a humidified atmosphere of 5% CO_2_. hFOB1.19 cells were cultured in DMEM/F12 supplemented with 0.3 mg/mL G418, 10% FBS and1% P/S (all purchased from Procell (Wuhan, China)), and maintained at 33.5 °C in a humidified atmosphere of 5% CO_2_. All cell lines were authenticated by STR sequencing and tested negative for mycoplasma contamination.

### Drugs, siRNAs, miRNA mimic and inhibitor

For RNA digestion, RNase R was purchased from Yeasen (Shanghai, China). The protein synthesis inhibitor cycloheximide (CHX) was purchased from Sigma (USA), and the proteasome inhibitor Z-Leu-Leu-Leu-al (MG132) was purchased from MCE (Shanghai, China). The siRNA target circ_0002669 and MYCBP were purchased from GenePharma (Shanghai, China). The negative control (NC), miR-889-3p mimic and inhibitor were synthesized by GenePharma (Supplemental Table 1).

### Plasmid transfection and stable cell line generation

To establish stable cell lines, the circ_0002669 sequence was cloned into the pLent-EF1a vector (Vigene, Guangzhou, China) and co-transfected with packaging vectors psPAX2 and pMD2G into HEK293T cells, for lentivirus production, using linear polyethylenimine (PEI, Yeasen, Shanghai, China) in accordance with the manufacturer’s instructions. To generate stable circ_0002669 knockdown OS cell lines, cells were lentivirally transduced with circ_0002669 shRNA (GenePharma, Shanghai, China), then selected in 10 µg/ml puromycin for one week. pcDNA3.1-MYCBP, HA-wildtype ubiquitin and K11, K48 and K63 mutants were purchased from Vigene (Guangzhou, China). Plasmids were transfected with Lipofectamine 3000 (Invitrogen, Carlsbad, CA, USA) in the indicated cells according to the manufacturer’s instructions.

### Western blotting and antibodies

RIPA buffer (Beyotime, Jiangsu, China) with protease inhibitor and phosphatase inhibitor cocktails (Beyotime) was used to extract protein lysates. Western blotting procedures were performed as described previously [19]. Antibodies against c-MYC (E5Q6W) (1:1000, #18,583), GAPDH (14C10) (1:1000, #2118), CCND1 (E3P5S) (1:1000, #55,506), c-Jun (60A8) (1:1000, #9165), AGO2 (C34C6) (1:1000, #2897), CDK4 (D9G3E) (1:1000, #12,790), caspase-3 (1:1000, #9662), cleaved caspase-3 (Asp175) (5A1E) (1:1000, #9664), PARP (46D11) (1:1000, #9532), ubiquitin (E6K4Y) (1:1000, #20,326) and HA (C29F4) (1:1000, #3724) were purchased from Cell Signaling Technology (CST, MA, USA). MYCBP (2E9) (1:1000, sc-517,020) was purchased from Santa Cruz (Cambridge, MA, USA). Secondary antibodies: anti-rabbit IgG, HRP-linked antibody (1:1000, #7074) and HRP-conjugated anti-mouse IgG, antibody (1:1000, #7076) were purchased from CST (USA).

### RNA extraction and real-time quantitative polymerase chain reaction (RT-qPCR)

Total RNA was extracted from cells and tissues using a FastPure Cell/Tissue Total RNA Isolation Kit (Vazyme, Guangzhou, China) according to the manufacturer’s instructions. Nuclear and cytoplasmic RNA was extracted using a nuclear and cytoplasmic fractionation kit (ECOTOP SCIENTIFIC Biotechnology, Guangzhou, China). RNA concentration was measured spectrophotometrically at an optical density of 260 nm. Approximately 10 µg of RNA was extracted, and 1 µg RNA was used for reverse transcription. Reverse transcription for mRNA and miRNAs was performed with a HiScript III 1st Strand cDNA Synthesis kit (Vazyme) or Mir-X miRNA First Strand synthesis kit (Takara) for miRNA using random primers according to the manufacturer’s instructions. PCR was subsequently performed with a 2×Taq PCR Mix kit (Absin, Shanghai, China), and the product was run on a 2% agarose gel. The running conditions for PCR were as follows: for activating the DNA polymerase, hot start was performed by incubating for 2 min at 94 °C, and then initiating cycling at 94 °C for 30 s, 60 °C for 30 s and 72 °C for 60s for a total of 30 cycles. RT-qPCR was performed using ChamQ SYBR qPCR Master Mix (Vazyme) and an Applied Biosystems PCR System (ABI 7500). The conditions for qPCR were as follows: for activating the DNA polymerase, hot start was performed by incubating for 30 s at 95 °C, and then cycling at 95 °C for 10 s and 60 °C for 30 s for a total of 40 cycles. The primer sequences for the analyzed genes are summarized in Supplementary Table 2.

### Immunohistochemistry (IHC) staining and ISH

IHC was performed as described previously [19]. Paraffin sections were used for staining. Antibodies used for IHC were as follows: Ki-67 (D3B5) (1:200, #12,202, CST), cleaved caspase 3 (35A1E) (1:100, #9664, CST) and MYCBP (2E9) (1:100, sc-517,020, Santa Cruz). Images were captured with an Olympus microscope (Japan).

For ISH, a digoxin-labeled circ_0002669 probe was synthesized by Boster (Wuhan, China). ISH was performed using an ISH kit (Boster) based on the manufacturer’s guidelines. Briefly, 4-mm thick tissue sections were baked for 1 h at 60 °C. After being deparaffinized and rehydrated, the samples were digested with 20 µg/mL proteinase K in pre-warmed 50 mM Tris for 10 min at 37 °C. The slides were subjected to hybridization with 20 µl of the control or circ_0002669 probe for 8 h at 40 °C. After washing and blocking, tissue samples were incubated with a 50 µl biotin-labeled anti-DIG reagent at 37 °C for 1 h, 50 µl SABC reagent at 37 °C for 20 min, and 50 µl biotinylated horseradish peroxidase at 37 °C for 20 min in turn. Finally, sections were incubated with 3, 3’diaminobenzidine (DAB) solution for 10 min at room temperature. For control, the enclosed negative control sense probe was applied.

For both IHC and ISH staining, the samples were evaluated and scored separately by two pathologists as follows. For staining percentage, 0 was given for no staining, and 1, 2, 3 and 4 were for 1–24%, 25–49%, 50–74% and over 75% positive, respectively. Staining intensity was scored as 0, negative; 1, weak; 2, moderate; and 3, strong. The final score was obtained by multiplying the percent positive score and staining intensity score, and a final score ≤ 6 was considered as low expression and final score > 6 defined as high expression.

### Cell counting Kit-8 (CCK8), and colony formation assays

OS cells were transfected with the indicated plasmids. After 48 h, 5 × 10^3^ cells were placed in a 96-well microplate for 4 days. To determine cell proliferation, 10 µl CCK8 reagent (LiJi, Shanghai, China) was added to each well, and cells were incubated for 2 h, then absorbance at 450 nm relative to a blank well was measured. Colony formation was assessed by plating 1 × 10^3^ cells in 6-well plate. After 14 days of culture, 1% crystal violet (Solarbio, Beijing, China) was used to stain the clones for 30 min. Clones were counted under a light microscope.

### Transwell and wound healing assay

After transfection, 2 × 10^5^ OS cells were cultured in the serum-free medium for 12–16 h and then suspended in serum-free medium. The cells were seeded into the upper chambers and 500 µL complete medium was added into the bottom chambers (Corning, NY, USA) for migration assays or Matrigel-coated camber for invasion assays. After 24 h, the cells on the lower compartment were fixed in 4% paraformaldehyde (Beyotime) and stained with crystal violet (Solarbio), then photographed and counted with an optical microscope (Olympus, Tokyo, Japan). For the wound healing assay, cells were seeded in 6-well plates at a density of 1 × 10^6^ cells per well. a straight scratch was made using a 200 µl pipette tip when the density reached approximately 100%. After washing with PBS, the loose cells were removed and cells were cultured with serum-free medium. Images were taken with an optical microscope (Olympus) each day for 2 days. ImageJ software was used to measure the relative wound areas.

### CircRNA pull-down assay and mass spectrometry

Circ_0002669 binding proteins were identified with a CircRNA-Protein Pull-Down kit (Focobio, Guangzhou, China) according to the user’s protocols. Briefly, 10^7^ OS cells were harvested and lysed with 500 µl lysis buffer. Lysates were incubated with 60 µl streptavidin agarose beads at 4 ^o^C for 2 h. Then, cell-bead complexes were pulled down overnight at 4 ^o^C with a biotin-labeled circ_0002669 probe (Focobio) targeting the junction site. After washing, the beads were boiled in SDS buffer for protein elution and western blotting. For identification of the binding proteins, mass spectrometry was performed by the BGI Company (Shenzhen, China).

### Dual-luciferase reporter assay

We constructed a circ_0002669 or MYCBP 3’UTR wildtype or mutant fragment and inserted it into the psiCHECK2 vector. OS cells were seeded in 96-well plates at 2 × 10^4^ per well, and then were co-transfected with miR-889-3p mimics or control mimic were co-transfected with the luc-circ_0002669 or MYCBP reporter plasmid. After 48 h, luciferase activity was measured using a Dual-Glo Luciferase Assay System (Promega, Shanghai, China). Briefly, after washing with PBS, cells were harvested and lysed in PLB buffer at room temperature for 15 min. Then 100 µl Reagent LARII was added to measure firefly luciferase activity. Subsequently, the Renilla luciferase activity was measured after adding Stop & GLO Reagent.

### RNA immunoprecipitation (RIP)

RIP was performed using a RIP kit (BersinBio, Guangzhou, China). Cells were lysed in 500 µl of lysis buffer supplemented with a protease and phosphatase inhibitor cocktail. Then, 20 µl of Protein A + G agarose beads was incubated with 2 g of MYCBP or IgG antibody at 4 ^o^C overnight. After washing twice with lysis buffer, antibody-coated beads were added to the cell lysates and rotated for 3 h. Beads were washed with lysis buffer three times and RNA was extracted using TRIzol.

### RNA fluorescence in situ hybridization (FISH) and immunofluorescence (IF)

FISH was performed using specific probes (Boster) for circ_0002669 or miR-889-3p and carried out according to the manufacturers’ instructions. OS cells were cultured on coverslips and fixed with 4% paraformaldehyde. After washing with PBS, cells were incubated with the indicated antibodies at 4 °C overnight and then incubated with FITC-labeled secondary antibodies (Beyotime) at 37 °C for 2 h. Cells were treated with the FISH probe in hybridization buffer (GenePharma) and then stained with DAPI (Beyotime). IF was performed as described previously [19] and images were acquired on a confocal microscope (Zeiss, Oberkochen, Germany).

### MiRNA prediction

The predicted miRNAs sponged by circ_0002669 were identified via CircInteractome (https://circinteractome.nia.nih.gov/) and ENCORI (https://starbase.sysu.edu.cn/). Predicted miRNAs that targeted the MYCBP 3’ untranslated region (3’UTR) were screened using ENCORI and Targetscan (http://www.targetscan.org/vert_72/). The overlapping miRNA candidates were examined for sponging by circ_0002669 and binding to MYCBP.

### In vivo tumor assay

For in vivo tumor formation, stable circ_0002669-overexpressing or control U2OS cells were resuspended in 1:1 PBS/Matrigel (Corning) solution at a density of 5 × 10^6^ cells in 100 µl and injected subcutaneously in the flanks of 4-week-old female BALB/C nude mice (*n* = 7 per group) (Guangdong Medical Laboratory Animal Center, Guangzhou, China). The mice were raised in the Laboratory Animal Center of Shantou University Medical College based on the national standard of laboratory animals. The mice were maintained under a 12-h light-dark cycle in a room at a temperature between 23 and 26 ^o^C and humidity between 40 and 70%. The size of the tumor was measured every week and volume was calculated according to the formula Volume = (long diameter x short diameter^2^)/2. Euthanasia by cervical dislocation was administered after four weeks, and tumors were isolated and weighed.

For in vivo metastasis, 2 × 10^6^ cells in 100 µl were injected into nude mice through the tail vein. Five weeks later, lungs were dissected, and metastatic nodules were counted under a microscope. All procedures were approved by the Institutional Animal Care and Use Committee of Shantou University Medical College (NO. SUMC2022-010).

### Statistical analysis

Experiments were performed at least three times independently, and statistical analyses were performed using SPSS 22.0 and GraphPad Prism. Survival analysis was assessed using Kaplan–Meier curves. The Student’s t-test and one-way ANOVA were used to assess differences in variables between groups. The correlation between different expressions was tested by the Pearson coefficient. P-values < 0.05 were considered to indicate statistical significance.

## Results

### Circ_0002669 is highly expressed in OS tissues and correlates with poor survival

**Fig. 1:**
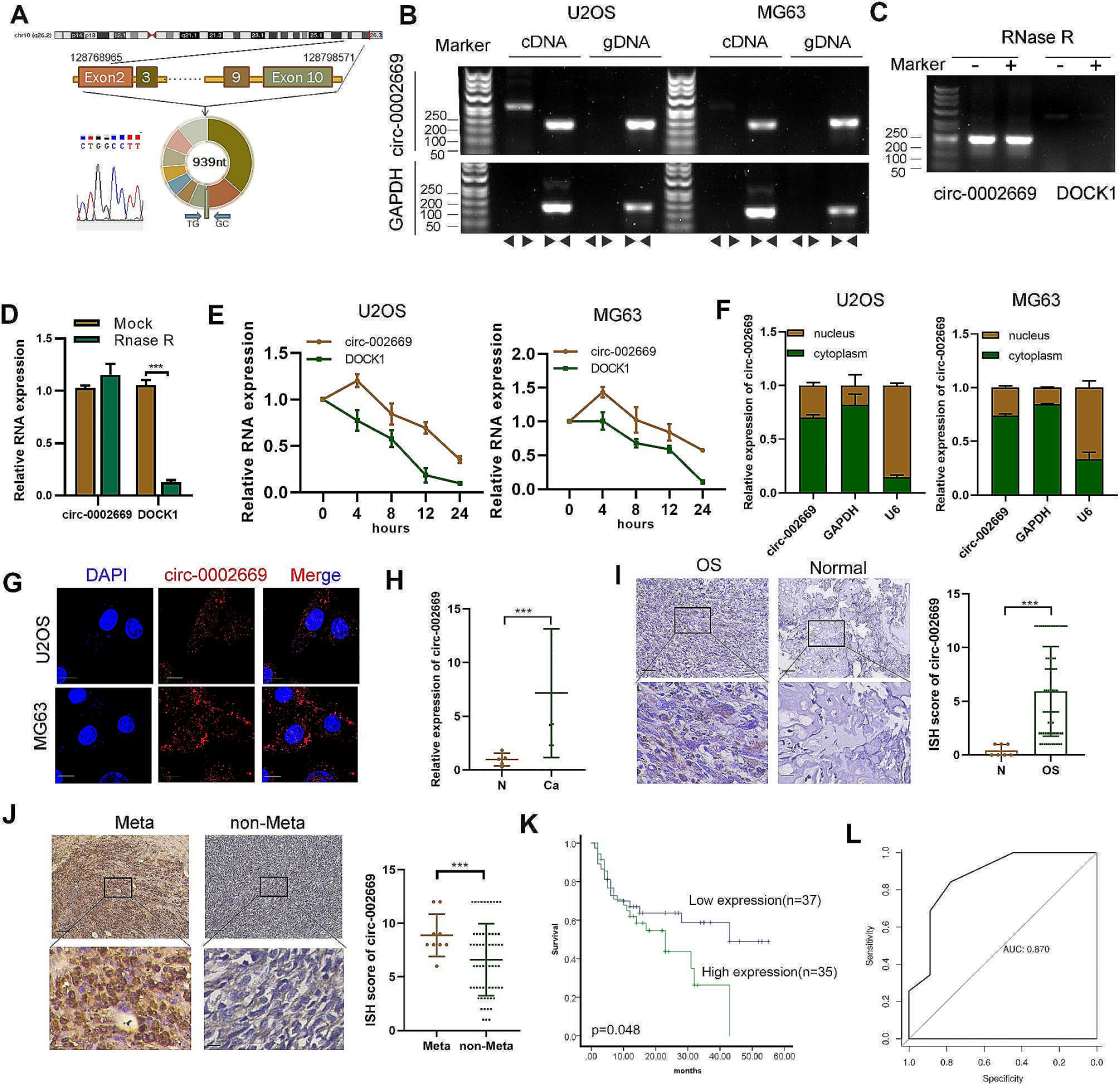
Verification of the structure and location of circ_0002669 and its clinical significance. **A** Circ_0002669 was generated from the DOCK1 gene located on chromosome 10. Sanger sequencing was used to identify the back-splice junction. **B** The closed loop structure of circ_0002669 was verified by PCR using convergent and divergent primers. **C, D** Upon RNase R treatment, expression of circ_0002669 and DOCK1 mRNA were detected by PCR and qRT-PCR in U2OS cells. **E** Circ_0002669 and DOCK1 mRNA expression were detected by qRT-PCR in OS cells treated with actinomycin D (1 μm) at the indicated time point. **F** Expression level of circ_0002669 in the nucleus and cytoplasm of cells was measured by qRT-PCR. **G** FISH was performed to identify the cellular location of circ_0002669 in OS cells (scale bar, 20 μm). **H** Circ_0002669 expression in 12 OS tissues and normal tissues (*n* = 5) was analyzed by qRT-PCR. (**I**) Circ_0002669 expression was detected in OS tissues and normal tissues by ISH (scale bar, 50 μm). **J** Circ_0002669 expression was detected in metastatic lesions and primary OS tissues (scale bar, 50 μm). **K** Survival analysis of 72 OS patients based on circ_0002669 ISH scores. **L** ROC curve based on the expression of circ_0002669 in OS tissues. Data shown are from three independent experiments, *p < 0.05, **p < 0.01, ***p < 0.001

Hsa_circ_0002669 is generated from the DOCK1 gene, located on chromosome 10 (chr10:128768965–128,798,571). The annotation for circ_0002669 includes exons 2 to 10 the DOCK1 gene (total 939 bp). By Sanger sequencing, the head-to-tail splicing of endogenous circ_0002669 was confirmed using divergent primers (Fig. 1A). The divergent primers for circ_0002669 amplified cDNA but not gDNA, indicating its circularity (Fig. 1B). Similarly, DOCK1 mRNA transcripts decreased whereas circ_0002669 was resistant to RNase R treatment, as determined by PCR and qPCR (Fig. 1C, D). Furthermore, circ_0002669 was more stable than DOCK1 in U2OS and MG63 cells treated with actinomycin D, a transcription inhibitor (Fig. 1E). After nuclear and cytoplasmic extraction, qRT-PCR revealed that circ_0002669 was localized in both the nucleus and cytoplasm, but mainly in the cytoplasm (Fig. 1F). RNA FISH confirmed this result (Fig. 1G).

Then we investigated the expression of circ_0002669 in cell lines and OS clinical samples. The expression of circ_0002669 was 6.5-fold higher in OS tissues (*n* = 12) than in normal tissues (*n* = 5) (Fig. 1H). The expression of circ_0002669 in cell lines (U2OS, HOS, Saos2 and MG63) was higher than in the hFOB1.19 cell line (Fig. S1A). Similarly, ISH staining showed circ_0002669 was 6.8-fold more highly expressed in OS tissue (*n* = 72) compared to normal tissue (*n* = 6) (Fig. 1I), and 1.4-fold higher in tissues with pulmonary metastases (*n* = 9) than in tissues without pulmonary metastases (*n* = 63) (Fig. 1J). Circ_0002669 expression had a tendency to positively, but not significantly correlate with lung metastasis (Supplemental Table 3). Kaplan–Meier analysis showed OS patients with higher circ_0002669 levels had unfavorable overall survival (Fig. 1K). ROC curve analysis gave an AUC of 0.87 (*p* < 0.01, Fig. 1L), indicating that circ_0002669 had good sensitivity and specificity as a diagnostic marker for OS. Taken together, these findings indicate that circ_0002669 is overexpressed in OS and correlates with poor survival.

### Circ_0002669 enhances the proliferation and migration of OS cells

**Fig. 2:**
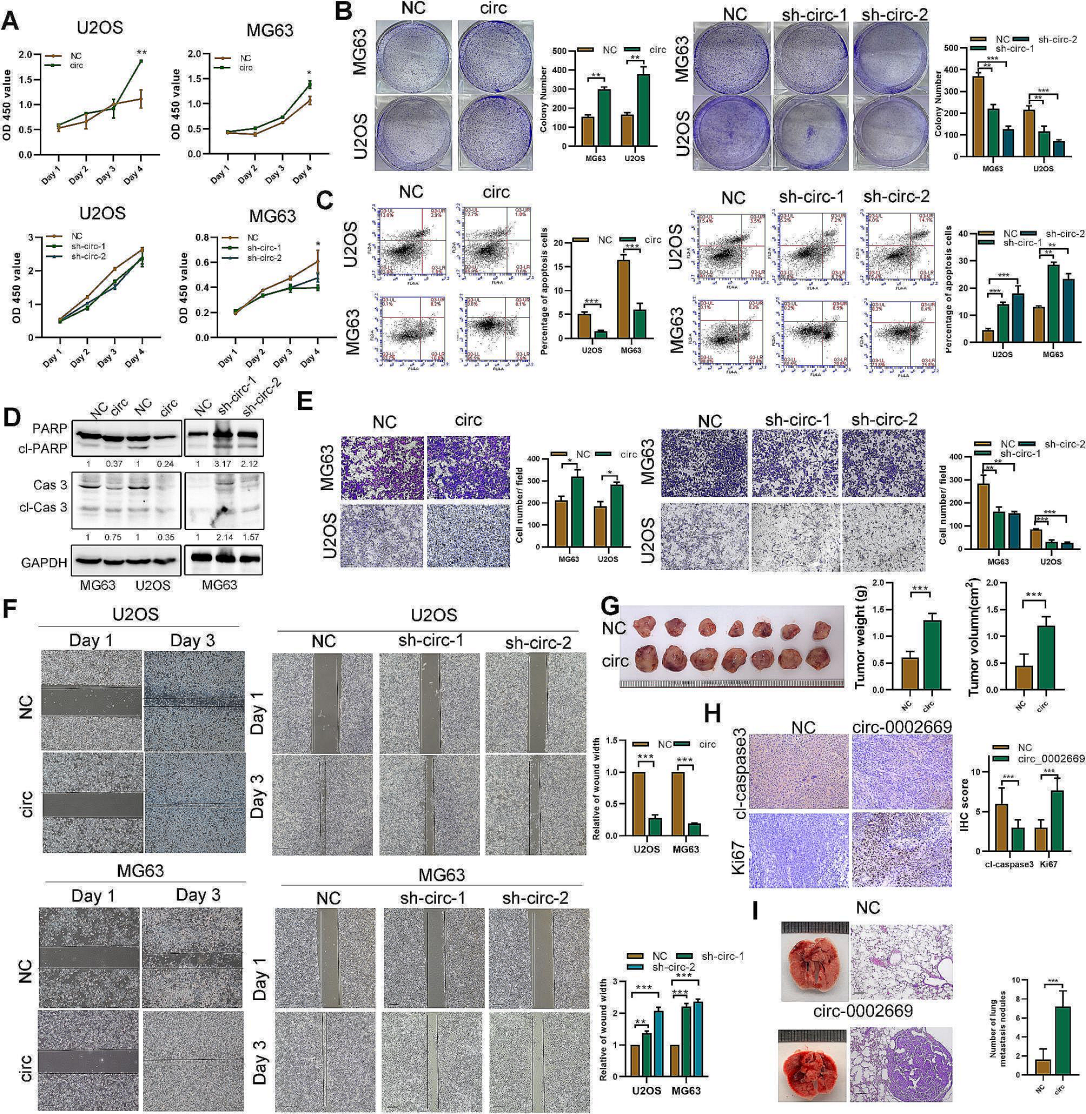
Circ_0002669 enhances OS cell proliferation and migration both in vitro and in vivo. **A** Cell proliferation was quantitated by CCK-8 assay. **B** Cell colony forming ability was measured. **C** Apoptosis of OS cells quantitated by flow cytometry via Annexin V-FITC/PI staining. **D** Expression of apoptosis-related proteins was detected using western blotting in OS cells. **E** Cell migration of OS cells determined by transwell assays (scale bar, 100 μm). **F** Migration of OS cells determined by wound healing assay. **G** Images of xenograft tumors in control and circ_0002669 groups (*n* = 7). **H** IHC staining of KI-67 and cleaved caspase-3 in control and circ_0002669 groups (scale bar, 50 μm). **I** Lung metastasis following tail vein injection of stable circ_0002669-overexpressing or control cells into nude mice. The mice were euthanized at 35 days. HE staining of lungs displayed metastatic nodules (scale bar, 50 μm). Data shown are from three independent experiments, *p < 0.05, **p < 0.01, ***p < 0.001

Considering the transfection efficiency and cell phenotype, we chose MG63 and U2OS as cell models and constructed stable circ_0002669-overexpressing cell lines and two stable circ_0002669 knockdown cell lines. The overexpression or knockdown efficiency was assessed by qRT-PCR (Fig. S2A). Overexpression of circ_0002669 did not affect DOCK1 mRNA levels (Fig. S2B), but enhanced OS cell proliferation, whereas circ_0002669 knockdown impaired proliferation, as determined by CCK-8 assays (Fig. 2A). Overexpression of circ_0002669 also enhanced the colony forming ability of OS cells (Fig. 2B), whereas knockdown of circ_0002669 augmented the spontaneous apoptosis of OS cells, as determined by flow cytometry (Fig. 2C). Western blotting also showed that circ_0002669 overexpression decreased the expression of cleaved caspase-3 and cleaved PARP (Fig. 2D). Evaluation of cell migration and invasion, by wound healing and transwell assays, indicated that knockdown of circ_0002669 prominently decreased, whereas circ_0002669 overexpression increased both cell migration and invasion (Fig. 2E, F and Fig. S2C).

To evaluate the effects of circ_0002669 on tumor growth and metastasis in vivo, stable circ_0002669-overexpressing cells were injected subcutaneously into the flank of 4-week-old female nude mice. Tumor volumes and weights in the circ_0002669-overexpressing group were larger and heavier than the control group (Fig. 2G). Consistently, in the circ_0002669-overexpressing group, IHC showed increases in Ki-67 expression, while the expression of cleaved caspase-3 was decreased compared with control group (Fig. 2H). In addition, circ_0002669-overexpressing cells, following injection into the tail vein of nude mice, formed more nodules in the lungs of nude mice, as detected by H&E staining (Fig. 2I). Taken together, these findings reveal circ_0002669 promotes the proliferation and migration of OS cells in vitro and in vivo.

### Circ_0002669 interacts with MYCBP to stabilize MYCBP expression

**Fig. 3:**
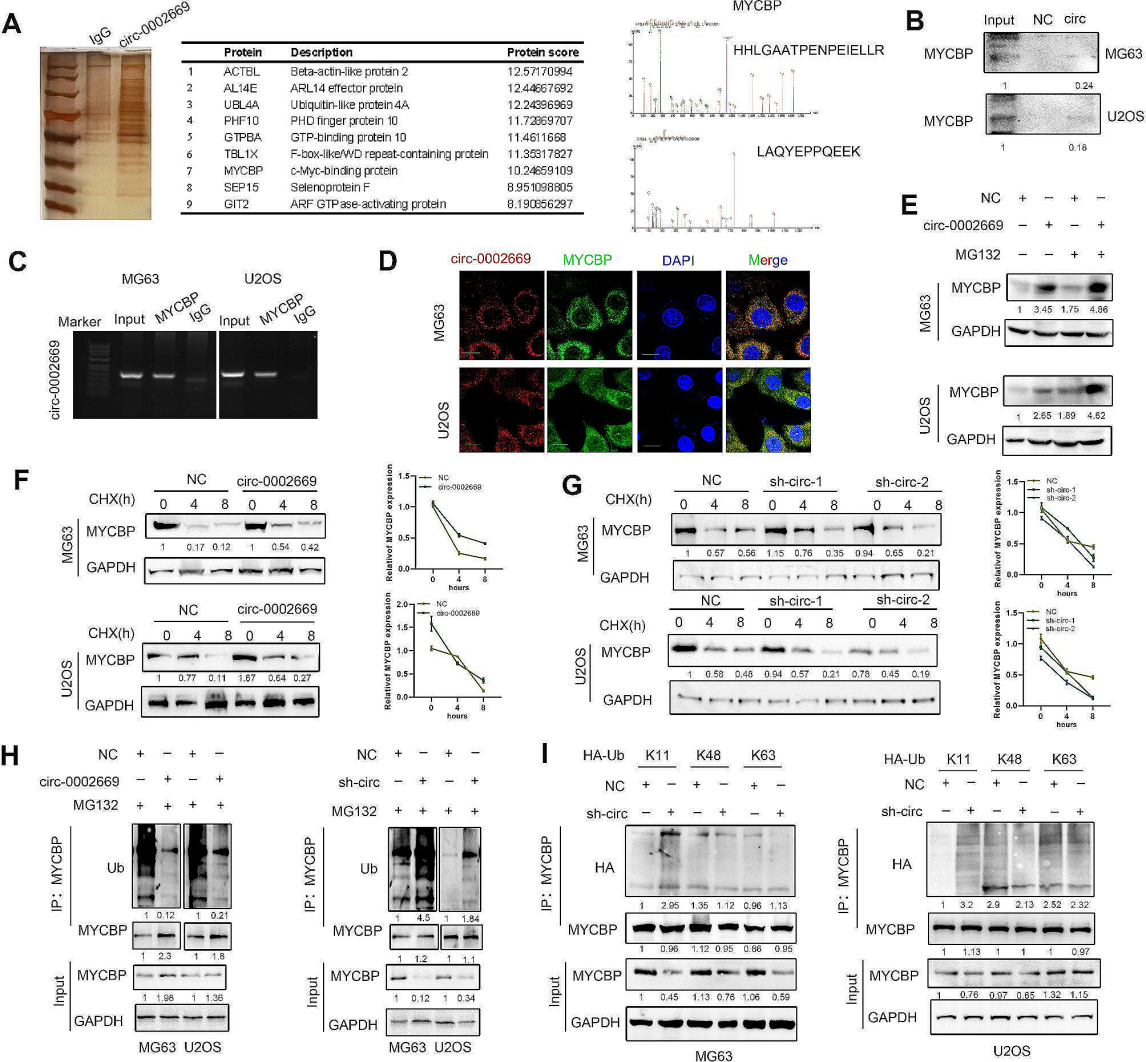
Circ_0002669 binds to MYCBP to upregulate MYCBP expression. **A** RNA pull-down was performed using the circ_0002669 biotin probe in U2OS cell lysates. After silver staining, MYCBP was identified as a candidate protein binding with circ_0002669 by mass spectrometry and western blot. **B** RNA pull-down was performed using biotin circ_0002669, and the relative expression of MYCBP was quantitated by western blotting. **C** RIP was performed on U2OS cell lysates using anti-MYCBP or anti-IgG, then the enrichment of circ_0002669 was detected by RT-PCR. **D** Circ_0002669 was co-localized with MYCBP in OS cells by FISH-IF (scale bar, 25 μm). **E** Stable circ_0002669-overexpressing or control OS cells were treated with MG132 (10 µM) and immunoblotted and probed with MYCBP antibody. **FG** Stable circ_0002669-overexpressing, -knockdown or control OS cells were treated with cycloheximide (CHX, 10 µM) and collected at different time points. Cell lysates were immunoblotted and probed with MYCBP antibody. **H** Ubiquitination of MYCBP in control, circ_002669-overexpressing or -knockdown OS cells were determined by immunoprecipitation with anti-MYCBP and then immunoblotting with the indicated antibodies. **I** Ubiquitination of MYCBP in OS cells, transfected with plasmids encoding different ubiquitin chains (K11, K48 and K63), was determined by immunoprecipitation with anti-MYCBP and then immunoblotting with the indicated antibodies. Data shown are from three independent experiments, *p < 0.05, **p < 0.01, ***p < 0.001

We further assessed the mechanisms through which circ_0002669 promotes OS malignancy. By RNA-biotin pulldown and mass spectrometry, we also found that MYCBP, a binding protein that stimulates c-MYC transcriptional activity, interacted with circ_0002669 (Fig. 3A and Supplemental Table 4). RIP analysis and biotin pulldown assay showed that circ_0002669 interacted with endogenous MYCBP in OS cells (Fig. 3B, C). FISH-immunofluorescence (IF) staining showed circ_0002669 co-localized with MYCBP in OS cells (Fig. 3D). MYCBP protein expression was also increased in circ_0002669-overexpressing cells in the presence of MG132, a proteasome inhibitor (Fig. 3E). To further investigate whether circ_0002669 regulates the protein stability of MYCBP, we treated circ_0002669-overexpressing or -knockdown OS cells with cycloheximide (CHX), a protein synthesis inhibitor, for different times. As shown in Fig. 3F, G, knockdown of circ_0002669 caused faster degradation of MYCBP in OS cells compared to control, while circ_0002669 overexpression protected MYCBP from degradation. After inhibiting the expression of circ_0002669 in OS cells, immunoprecipitation of ubiquitin showed that the level of MYCBP protein ubiquitination was enhanced. As expected, MYCBP ubiquitination was substantially decreased when circ_0002669 was overexpressed (Fig. 3H). Furthermore, we found that circ_0002669 controlled MYCBP ubiquitination and degradation through K11 chain linkage (Fig. 3I). These data indicate that circ_0002669 interacts with MYCBP to protect it from protein degradation in OS.

### Circ_0002669 serves as a miR‑889‑3p sponge to regulate MYCBP mRNA expression

**Fig. 4:**
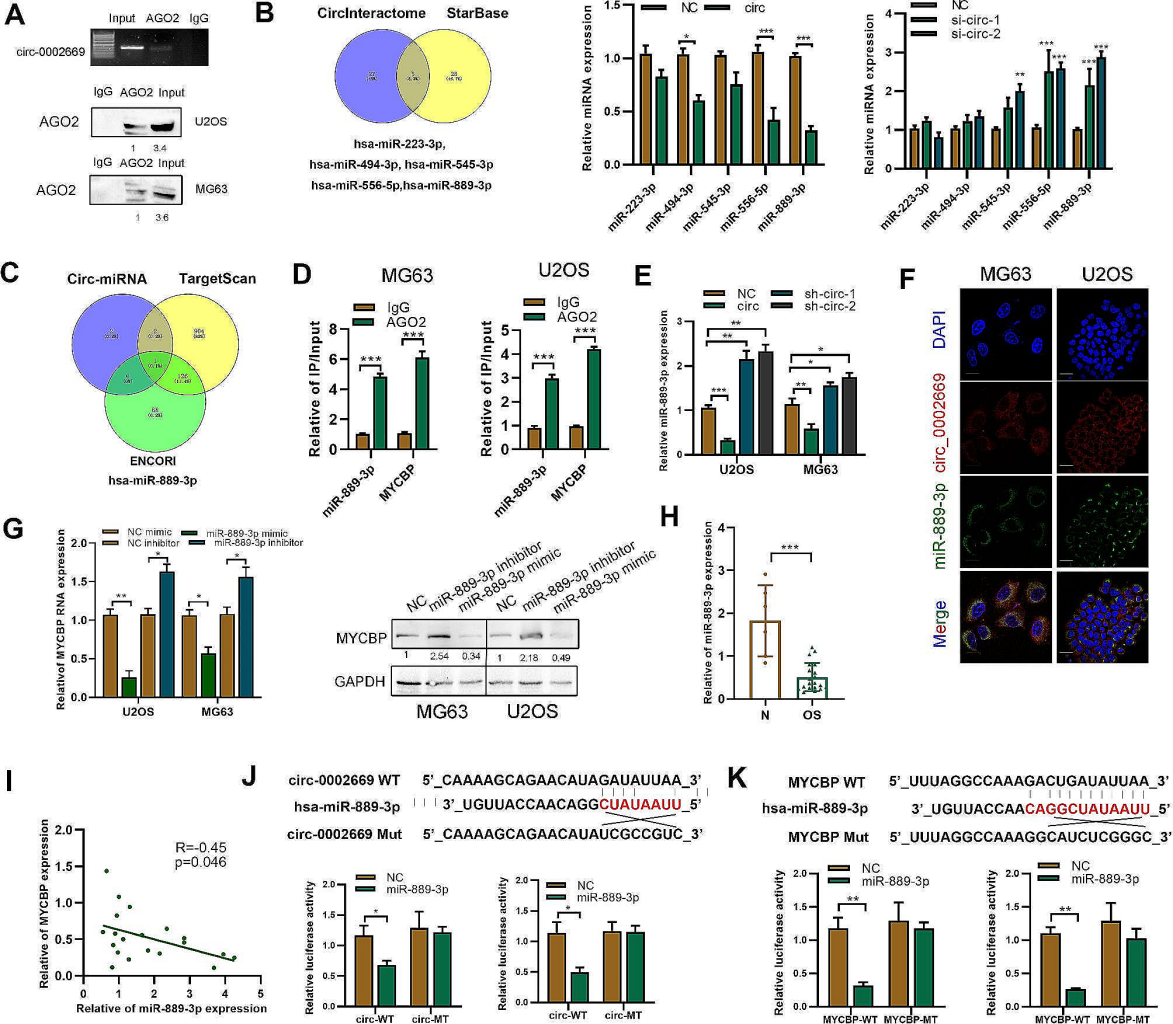
Circ_0002669 functions as a sponge for miR-889-3p. **B** RIP and RT-PCR was performed using U2OS cells to determine the enrichment of circ_0002669. RNA pull-down was performed, followed by western blotting to determine AGO2 expression. **B** The potential miRNAs binding to circ_0002669, as predicted by StarBase and CircInteractome. Expression of the miRNAs was determined in circ_0002669-overexpressing or -knockdown OS cells. **C** Identification of miR-889-3p by using TargetScan and ENCORI to screen for upstream miRNAs of MYCBP. **D** RIP was performed to detect the binding between miR-889-3p or MYCBP mRNA and AGO2 protein. **E** MiR-889-3p expression was determined using qRT-PCR in circ_0002669-overexpressing or -knockdown OS cells. **F** FISH was performed to determine the intracellular location of circ_0002669 (red) and miR-889-3p (green) (scale bar, 20 μm). **G** Expression of MYCBP was determined in miR-889-3p mimic- or inhibitor-transfected OS cells, as determined by qRT-PCR and western blotting. **H** Relative expression of miR-889-3p in OS tissues (*n* = 12) and non-tumor tissues (*n* = 5) was determined by qRT-PCR. **I** Correlation between miR-889-3p and MYCBP expression in OS tissues. **J** Schematic illustration of circ_002669-wildtype (WT) and circ_002669-Mut (MT) luciferase reporter vectors. Relative luciferase activities were determined in the indicated transfected OS cells. **K** Schematic illustration of MYCBP-WT and MYCBP-MT luciferase reporter vectors. The relative luciferase activities were quantitated in the indicated transfected OS cells. Data shown are from three independent experiments, *p < 0.05, **p < 0.01, ***p < 0.001

We found circ_0002669 not only enhanced MYCBP protein expression, but also mRNA expression (Fig. S3A). Thus, we explored the possible mechanisms. We found that circ_0002669 was localized in the cytoplasm and could interact with AGO2, as predicted by CircInteractome (https://circinteractome.nia.nih.gov/), indicating that circ_0002669 could work as a ceRNA (Fig. S3B). By circRNA-biotin pulldown and RIP, circ_0002669 was found to be enriched in AGO2 precipitate compared with IgG precipitate (Fig. 4A). To determine whether circ_0002669 could function as a miRNA sponge, we identified five miRNAs (hsa-miR-494-3p, hsa-miR-223-3p, hsa-miR-545-3p, hsa-miR-889-3p, hsa-miR-556-5p) as putative targets of circ_0002669, by using StarBase and CircInteractome, then detected their expression in control and circ_0002669-overexpressing or -knockdown OS cells (Fig. 4B). Next, using TargetScan and ENCORI to predict upstream miRNAs of MYCBP, we identified miR-889-3p as having the same miRNA response binding elements of MYCBP and circ_0002669 (Fig. 4C and S3C). RIP assays further indicated that both miR-889-3p and MYCBP mRNA were enriched in the AGO2 group, but not in the IgG group in OS cells (Fig. 4D). As shown in Fig. 4E, miR-889-3p was downregulated after circ_0002669 transfection, but upregulated after circ_0002669 knockdown, compared with the scrambled control. Co-localization experiments showed circ_0002669 and miR-889-3p both co-localized in the cytoplasm (Fig. 4F). The expression level of MYCBP was also decreased after transfection of a miR-889-3p mimic, and increased by transfection of a miR-889-3p inhibitor in both OS cell lines, as determined by western blotting and qRT-PCR (Fig. 4G and S3D). Then, we investigated the expression of miR-889-3p in human OS tissues. As shown in Fig. 4H, miR-889-3p expression was 3.7-fold decreased in OS tissues when compared to normal tissues. In addition, MYCBP and miR-889-3p showed potential correlation in OS samples (Fig. 4I).

Bioinformatic analysis revealed that circ_0002669 shared miRNA response elements with miR-889-3p, so we constructed and cloned both wildtype (WT) and mutant miRNA response elements into a luciferase reporter containing the circ_0002669 sequence and found that the luciferase activities of the mutant reporter were significantly higher than the WT reporter after miR-889-3p transfection, indicating that miR-889-3p can directly bind to circ_0002669 (Fig. 4J). Similarly, luciferase activity was markedly decreased after transfection with WT vector and the miR-889-3p mimic, but unchanged by the mutated vector, indicating miR-889-3p may directly bind to the 3′-UTR of MYCBP mRNA (Fig. 4K). These results suggest that circ_0002669 upregulates MYCBP mRNA expression by inhibiting miR-889-3p in OS.

### Circ_0002669 increases c-MYC transcriptional activity

**Fig. 5:**
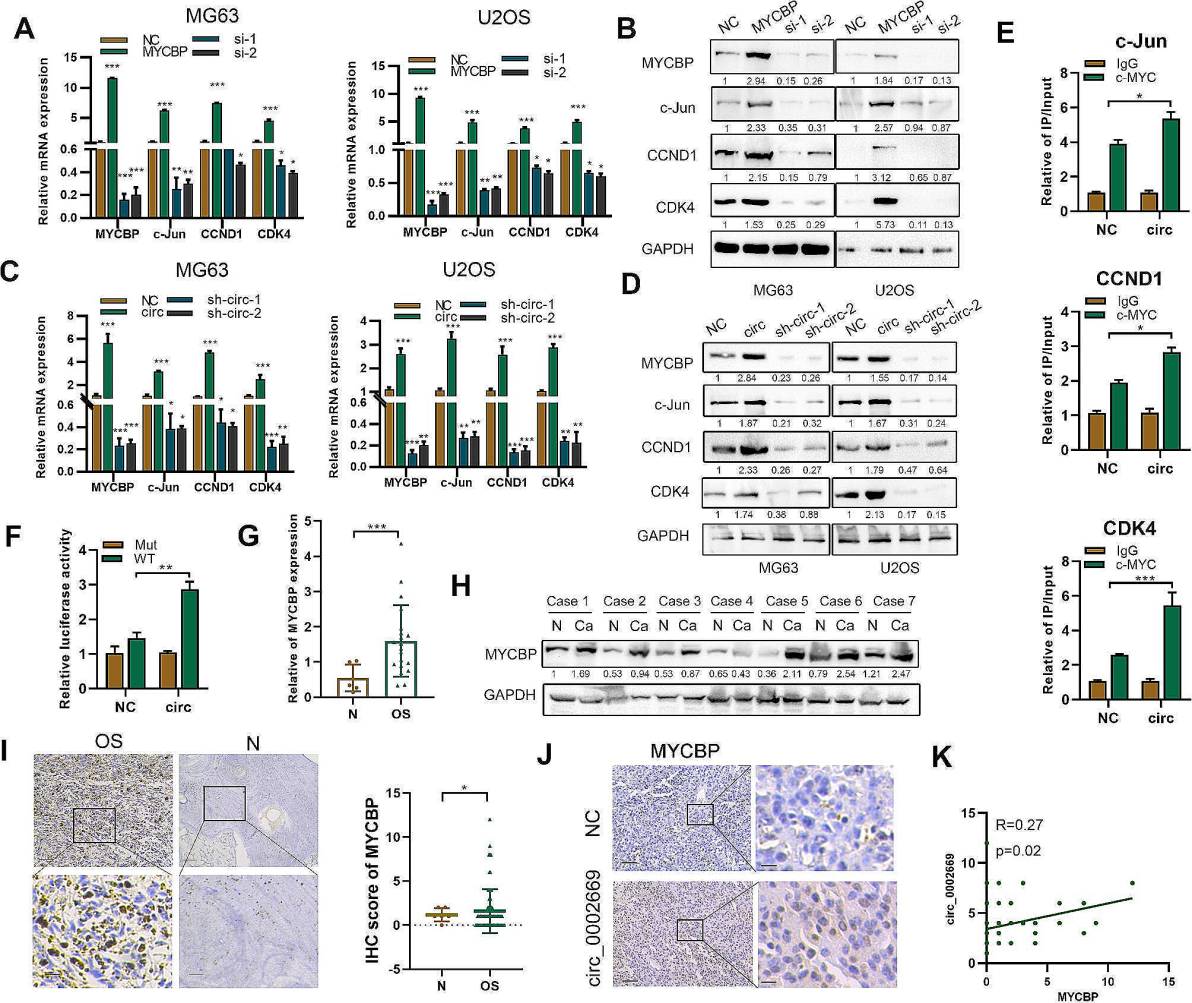
Circ_0002669 increases c-MYC transcriptional activity. **A, B** Relative expression of MYCBP and c-MYC downstream genes was determined, by qRT-PCR and western blotting, in OS cells transfected with MYCBP plasmid or siRNAs. **C, D** Relative expression of MYCBP and c-MYC downstream genes was determined in circ_0002669-overexpressing or -knockdown OS cells by qRT-PCR and western blotting. **E** CHIP-qPCR showing circ_002669 enhanced c-MYC occupancy on the c-Jun, CCND1 and CDK4 promoters in U2OS cells. **F** Circ_002669 in U2OS cells increased transcription of the c-Jun promoter. **G** MYCBP expression in OS (*n* = 12) and normal tissues (*n* = 5) was determined by qRT-PCR. **H** MYCBP expression in OS and normal tissues (*n* = 7) was determined by western blotting. **I** MYCBP expression in OS tissues (*n* = 72) and normal tissues (*n* = 6) was determined by IHC. **J** MYCBP expression in control or circ_002669-overexpressing samples derived from xenografts (scale bar, 100 μm). **K** Pearson correlation analysis showing the correlation between expression of MYCBP and circ_002669 in OS tissues. Data shown are from three independent experiments, *p < 0.05, **p < 0.01, ***p < 0.001

MYCBP can enhance the expression and transcription of c-MYC downstream genes, such as CCND1, c-Jun and CDK4 [14]. The expression of these genes was increased in MYCBP-overexpressing cells compared to control cells (Fig. 5A, B). Furthermore, the expression of these genes was upregulated by circ_0002669 overexpression, and decreased by circ_0002669 knockdown (Fig. 5C, D), and circ_0002669 increased c-MYC transcriptional activity, as detected by luciferase reporter assay and CHIP-qPCR (Fig. 5E, F).

Then, we examined MYCBP expression by western blotting, qRT-PCR and immunohistochemistry. Similar to circ_0002669 expression, MYCBP expression was 2.73-fold higher in OS tissues than normal tissues (Fig. 5G-I). High MYCBP staining was displayed in high circ_0002669-expressing tumors generated from xenografts (Fig. 5J). Importantly, MYCBP protein expression had a positive tendency to correlate with circ_0002669 expression in OS (*R* = 0.27, *p* = 0.02) (Fig. 5K). Collectively, these results demonstrate that circ_0002669 increases transcriptional activity of c-MYC and correlates with MYCBP expression in OS.

### Circ_0002669 regulates OS malignancy via a miR-889-3p/MYCBP axis

**Fig. 6:**
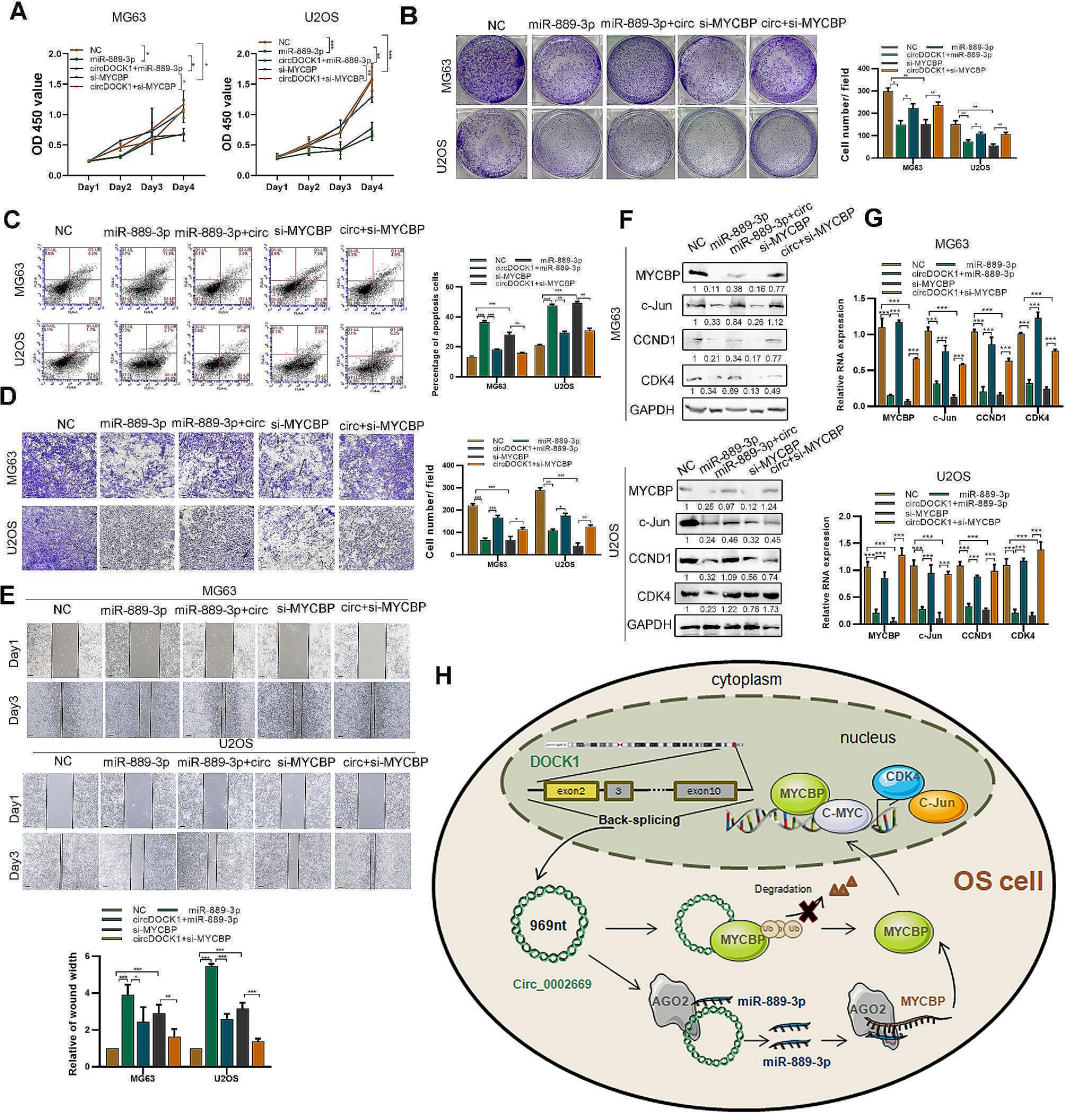
Circ_0002669 promotes OS malignancy through a miR-889-3p/MYCBP axis. **A, B** Cell proliferation and colony formation was determined by CCK-8 and colony formation assays in control, miR-889-3p, miR-889-3p + circ_0002669, si-MYCBP and si-MYCBP + circ_0002669 OS cell subgroups. **C** Apoptosis was measured by flow cytometry following annexin V-FITC/PI staining in control, miR-889-3p, miR-889-3p + circ_0002669, si-MYCBP and si-MYCBP + circ_0002669 OS cell groups. **D, E** Cell migration was determined by transwell and wound healing assays in control, miR-889-3p, miR-889-3p + circ_0002669, si-MYCBP and si-MYCBP + circ_0002669 OS cell groups (scale bar, 100 μm). **F, G** Relative expression of MYCBP, c-Jun, CCND1 and CDK4 mRNA and protein levels was determined by qRT-PCR and western blotting in control, miR-889-3p, miR-889-3p + circ_0002669, si-MYCBP and si-MYCBP + circ_0002669 OS cell groups. **H** Schematic diagram of this paper. Data shown are from three independent experiments, *p < 0.05, **p < 0.01, ***p < 0.001

We then investigated whether circ_0002669 regulated OS malignant progression by decreasing miR-889-3p expression or increasing MYCBP expression, we transfected a miR-889-3p mimic or si-MYCBP into stable circ_0002669-overexpressing OS cells. As shown in Fig. 6A, B, the miR-889-3p mimic or MYCBP knockdown abolished the positive effects of circ_0002669 on cell proliferation and colony formation, and reversed the inhibitory effects of circ_0002669 on cell apoptosis (Fig. 6C). The cell migration induced by circ_0002669 overexpression in U2OS and MG63 cells was also inhibited by MYCBP knockdown or the miR-889-3p mimics (Fig. 6D, E). Furthermore, the increased expression of c-MYC downstream genes induced by circ_0002669 was partially decreased by incubation with MYCBP siRNAs or the miR-889-3p mimic (Fig. 6F, G). These results indicate that circ_0002669 promotes OS malignancy and mediates c-MYC transcriptional activity through the regulation of miR-889-3p or MYCBP expression.

## Discussion

In our study, we found that circ_0002669 is upregulated in OS and correlates with poor survival. Circ_0002669 enhances, while circ_0002669 knockdown reduces OS cell proliferation and migration in OS cell lines. We also found that circ_0002669 plays an important role as a ceRNA of miR-889-3p to regulate MYCBP expression. Moreover, circ_0002669 could bind to MYCBP and protect it from proteasome degradation, which has tremendous effects on the growth and migration of OS cells (Fig. 6H).

Interestingly, accumulating reported evidence indicates that DOCK1 is a key oncogene that drives tumorigenesis by mediating cell migration and metastasis, such as liver cancer and breast cancer [10, 11]. Several circRNAs generated via back-splicing of the 3’ and 5’ ends of different exons of DOCK1 have been identified and explored. CircDOCK1 (circ_0020394) promotes the progression of breast cancer via regulating the miR-132-3p/Sox5 pathway [20]. CircDOCK1 (circ_0007142) upregulates CDC25A expression to promote colorectal cancer progression by sponging miR-122-5p [21]. CircDOCK1 (circ_0020378) promotes OS progression and chemotherapy sensitivity via miR-339-3p/IGF1R and miR-936/LEF1pathways [22, 23]. Interference with circ-DOCK1 (circ_0020397) inhibits hepatocellular carcinoma cell proliferation, invasion and migration via the miR‑654‑5p/SMAD2 axis [24]. In our paper, Sanger sequencing revealed a novel circRNA, denoted circ_0002669, that is generated from *DOCK1* exons 2 to 10.

We show circ_0002669 expression is upregulated in OS tissues and cells, as determined by qPCR and ISH. Analysis of the clinicopathological characteristics of 72 OS patients revealed that circ_0002669 expression has a positive tendency to correlate with lung metastasis, although it is not significant. We believe that statistical significance may be achieved with an increase in the sample size. We also demonstrated a functional role of circ_0002669 in promoting the proliferation and invasion of OS cells in vivo and in vitro.

Mechanistically, circRNAs modulate gene expression by acting as miRNA sponges, regulating endogenous gene transcription and interacting with RNA-binding proteins [25]. Thus, we explored the mechanisms by which circ_0002669 promotes OS tumorigenesis and identified MYCBP as a circ_0002669-binding protein in OS cells. MYCBP is normally found in the cytoplasm, but translocates into the nucleus and binds with c-MYC through its C-terminal domain. The MYCBP/c-MYC pathway plays an important role in tumor development [14]. MYCBP was identified as a target of β-catenin/lymphoid enhancer-binding factor (LEF) transcriptional regulation in colon carcinoma [26]. Patients with low MYCBP expression have better survival in low grade glioma and hepatocellular carcinoma [15, 16]. MYCBP is upregulated in OS tissues compared with normal tissues. It has been reported that the expression of MYCBP protein stability is mediated by sperm-associated antigen 5 (SPAG5) in a proteasome degradation-dependent manner [27], thus we hypothesized that circ_0002669 could also mediate MYCBP protein stability. RIP, biotin pulldown and FISH-IF assays further validated the binding of circ_002669 to MYCBP. We observed that circ_0002669 increases both protein expression and stability of MYCBP. Moreover, a significant correlation was observed between circ_002669 and MYCBP protein expression in OS.

Cytoplasmic circRNAs can bind the AGO2 protein to sponge miRNAs and induce the degradation of the target mRNAs [28]. We found that cytoplasmic circ_0002669 can bind AGO2, suggesting that circ_0002669 might work as a miRNA sponge. As predicted by bioinformatics databases, five candidate miRNAs were identified. Among these miRNAs, miR-889-3p was shown to combine with circ_0002669 by luciferase reporter assay and RNA pulldown. Collectively, our results show circ_0002669 directly targets miR-889-3p to increase MYCBP expression in OS cells. The expression and function of miR-889-3p in the pathogenesis of multiple tumors has been demonstrated. However, the findings are not without controversy. In lung cancer, miR-889-3p inhibits cell proliferation and EMT by downregulating homeodomain interacting protein kinase 1 (HIPK1) [29]. In OS, miR-889-3p could enhance cell proliferation through inhibiting the expression of myeloid cell nuclear differentiation antigen (MNDA) [30]. In this study, we found that circ_0002669 decreases miR-889-3p expression while increasing MYCBP expression. Moreover, our functional rescue experiments showed that re-expression of miR-889-3p or knockdown MYCBP abolishes circ_0002669-induced OS cell growth and invasion. Furthermore, MYCBP expression is negatively correlated with miR-889-3p expression in OS patient samples. Taken together, we identify a circ_0002669/miR-889-3p/MYCBP regulatory network promotes OS tumorigenesis.

## Conclusion

Our study demonstrates that circ_0002669 promotes OS cell proliferation and migration via binding with MYCBP and regulates the miR-889-3p/MYCBP axis, which may provide a novel mechanism for the pathogenesis of OS and a therapeutic target in the treatment of OS.

### Electronic supplementary material

Below is the link to the electronic supplementary material.

Supplementary Material 1

Supplementary Material 2

Supplementary Material 3

Supplementary Material 4

Supplementary Material 5

Supplementary Material 6

Supplementary Material 7

## Data Availability

No datasets were generated or analysed during the current study.

## Abbreviations

3’-UTR: 3’-untranslated region
CCK-8: Cell Counting Kit-8
CCND1: Cyclin D1
CDK4: Cyclin-dependent kinase 4
CHX: Cycloheximide
ChIP: Chromatin immunoprecipitation
circRNA: Circular RNA
DAPI: 4,6-Diamidino-2-phenylindole
DEG: Differentially-expressed gene
DMEM: Dulbecco’ s modified Eagle’s medium
DOCK: Dedicator of cytokinesis
EMT: Epithelial-mesenchymal transition
FBS: Fetal bovine serum
GAPDH: Glyceraldehyde-3-phosphate dehydrogenase
GO: Gene Ontology
IHC: Immunohistochemistry
IP: Immunoprecipitation
MG132: Carbobenzoxy-Leu-Leu-leucinal
MYCBP: C-MYC binding protein
NC: Negative control
OS: Osteosarcoma
PBS: Phosphate-buffered saline
qRT-PCR: Quantitative reverse transcription-polymerase chain reaction
RIP: RNA immunoprecipitation
