# Supplementary material for: Circ_0002669 promotes osteosarcoma tumorigenesis through directly binding to MYCBP and sponging miR-889-3p
Source: Biol Direct. 2024 Apr 3;19:25. doi: 10.1186/s13062-024-00466-1 (PMC10988859; doi:10.1186/s13062-024-00466-1)
Supplement: Supplementary file 1 — Supplementary Material 1 [file 13062_2024_466_MOESM1_ESM.docx]

|  | **Protein** | **Description** | **Protein score** |
| --- | --- | --- | --- |
| 1 | ACTBL | Beta-actin-like protein 2 | 12.57170994 |
| 2 | AL14E | ARL14 effector protein | 12.44667692 |
| 3 | UBL4A | Ubiquitin-like protein 4A | 12.24396969 |
| 4 | PHF10 | PHD finger protein 10 | 11.72869707 |
| 5 | GTPBA | GTP-binding protein 10 | 11.4611668 |
| 6 | TBL1X | F-box-like/WD repeat-containing protein | 11.35317827 |
| 7 | MYCBP | c-Myc-binding protein | 10.24659109 |
| 8 | SEP15 | Selenoprotein F | 8.951098805 |
| 9 | GIT2 | ARF GTPase-activating protein | 8.190856297 |

**Supplemental table 4. List of possible circ_0002669-interacting protein identified by mass spectromatry**
