# Supplementary material for: Circ_0002669 promotes osteosarcoma tumorigenesis through directly binding to MYCBP and sponging miR-889-3p
Source: Biol Direct. 2024 Apr 3;19:25. doi: 10.1186/s13062-024-00466-1 (PMC10988859; doi:10.1186/s13062-024-00466-1)
Supplement: Supplementary file 2 — Supplementary Material 2 [file 13062_2024_466_MOESM2_ESM.docx]

| **Oligo names** | **Sequences (5’ ́-3’ ́)** |
| --- | --- |
| miR-889-3p mimic | UUAAUAUCGGACAACCAUUGU |
| miR-889-3p inhibitor | ACAAUGGUUGUCCGAUAUUAA |
| Si-MYCBP-1 | CUGUCACUAUGGCCCAUUATT |
| Si-MYCBP-2 | UGGCCGAAAUGAAAGAGAATT |
| si-control | UUCUCCGAACGUGUCACGUTT |
| Control mimic | UUCUCCGAACGUGUCACGUTT |
| Control inhibitor | CAGUACUUUUGUCUAGUACAA |
| sh-circ_0002669-1 | GGCUGCUUUUUAUAACUAUGATT |
| sh-circ_0002669-2 | GUGGCUGCUUUUUAUAACUAUTT |
| sh-control | UUCUCCGAACGUGUCACGUTT |

**Supplemental Table 1. Sequences of synthesized mimic inhibitor probes used in this study**
