# Supplementary material for: Circ_0002669 promotes osteosarcoma tumorigenesis through directly binding to MYCBP and sponging miR-889-3p
Source: Biol Direct. 2024 Apr 3;19:25. doi: 10.1186/s13062-024-00466-1 (PMC10988859; doi:10.1186/s13062-024-00466-1)
Supplement: Supplementary file 3 — Supplementary Material 3 [file 13062_2024_466_MOESM3_ESM.docx]

| **Parameters** |  | **circ _0002669 expression** | | **Number** | ***P*** |
| --- | --- | --- | --- | --- | --- |
|  |  | **Low(n=37)** | **High(n=35)** |  |  |
| **Age** | >25 | 11 | 18 | 29 | 0.092 |
|  | ≤25 | 26 | 17 | 43 |  |
| **Gender** | Male | 24 | 24 | 48 | 0.806 |
|  | Female | 13 | 11 | 24 |  |
| **Location*** | Tibia | 11 | 10 | 21 | 0.21 |
|  | Fibula | 1 | 2 | 3 |  |
|  | Thighbone | 17 | 9 | 26 |  |
|  | Other | 8 | 14 | 22 |  |
| **Lung Metastasis** | Yes | 2 | 7 | 9 | 0.081 |
|  | No | 35 | 28 | 63 |  |
| **Invasion** | Yes | 3 | 6 | 9 | 0.301 |
|  | No | 34 | 29 | 63 |  |
| **Tumor size** | >4 cm | 21 | 21 | 42 | 0.815 |
|  | <4 cm | 16 | 14 | 30 |  |
| **Lymph node metastasis** | Yes | 2 | 2 | 4 | 1 |
|  | No | 35 | 33 | 68 |  |

**Supplemental Table 3. The correlation between circ_0002669 expression and OS clinical parameters**

*Tibia vs Fibula: p=1.0; Tibia vs Thighbone: p=0.3896; Tibia vs other:p=0.364;

Fibula vs Thighbone: p=0.54; Fibula vs other:p=1; Thighbone vs other:p=0.08
