# Supplementary material for: Circ_0002669 promotes osteosarcoma tumorigenesis through directly binding to MYCBP and sponging miR-889-3p
Source: Biol Direct. 2024 Apr 3;19:25. doi: 10.1186/s13062-024-00466-1 (PMC10988859; doi:10.1186/s13062-024-00466-1)
Supplement: Supplementary file 4 — Supplementary Material 4 [file 13062_2024_466_MOESM4_ESM.docx]

| **Gene name** | **Forward Primer** | **Reverse Primer** |
| --- | --- | --- |
| CCND1 | GCTGCGAAGTGGAAACCATC | CCTCCTTCTGCACACATTTGAA |
| CDK4 | ATGGCTACCTCTCGATATGAGC | CATTGGGGACTCTCACACTCT |
| c-Jun | ATGACTGCAAAGATGGAAACGACC | GATGTGCCCGTTGCTGGACTGGAT |
| hsa-miR-223-3p | GCGCGTGTCAGTTTGTCAAAT | AGTGCAGGGTCCGAGGTATT |
| hsa-miR-494-3p | CGCGTGAAACATACACGGGA | AGTGCAGGGTCCGAGGTATT |
| hsa-miR-545-3p | GCGCGTCAGCAAACATTTATT | AGTGCAGGGTCCGAGGTATT |
| hsa-miR-556-5p | CGCGGATGAGCTCATTGTAA | AGTGCAGGGTCCGAGGTATT |
| hsa-miR-889-3p | GCGCGTTAATATCGGACAAC | AGTGCAGGGTCCGAGGTATT |
| Circ-0002669 | CAGATTGTTCGCGTGGGTCG | GGTCACCCGGGATGACTGTT |
| MYCBP | ATGGCCCATTACAAAGCCG | TTTCTGGAGTAGCAGCTCCTAA |
| DOCK1(qPCR) | ACCGAGGTTACACGTTACGAA | TCGGAGTGTCGTGGTGACTT |
| DOCK1(PCR) | AACTTCTCCGGACCGTGAAC | AGATGGGGATGGTGGCTTTG |
| GAPDH | CATGTTCCAATATGATTCCACC | CACTTGATTTTGGAGGGATCTC |
| U6 | TGGAACGCTTCACGAATTTGCG | GGAACGATACAGAGAAGATTAGC |

**Supplemental Table 2. Primers**
