# Supplementary figures and images for: Circ_0002669 promotes osteosarcoma tumorigenesis through directly binding to MYCBP and sponging miR-889-3p
Source: Biol Direct. 2024 Apr 3;19:25. doi: 10.1186/s13062-024-00466-1 (PMC10988859; doi:10.1186/s13062-024-00466-1)

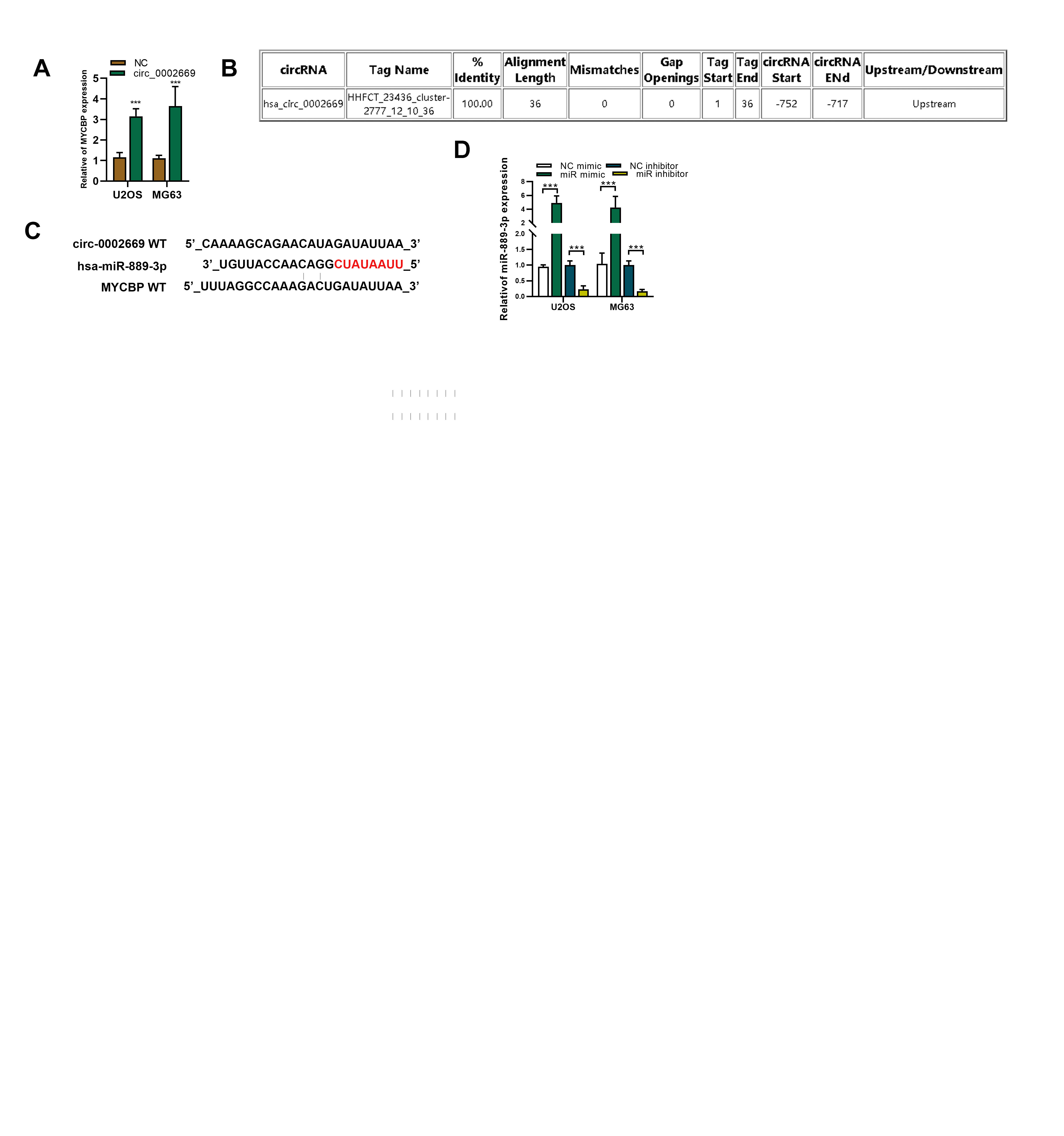

Supplement: Supplementary file 5 — Supplementary Material 5 [file 13062_2024_466_MOESM5_ESM.jpg]

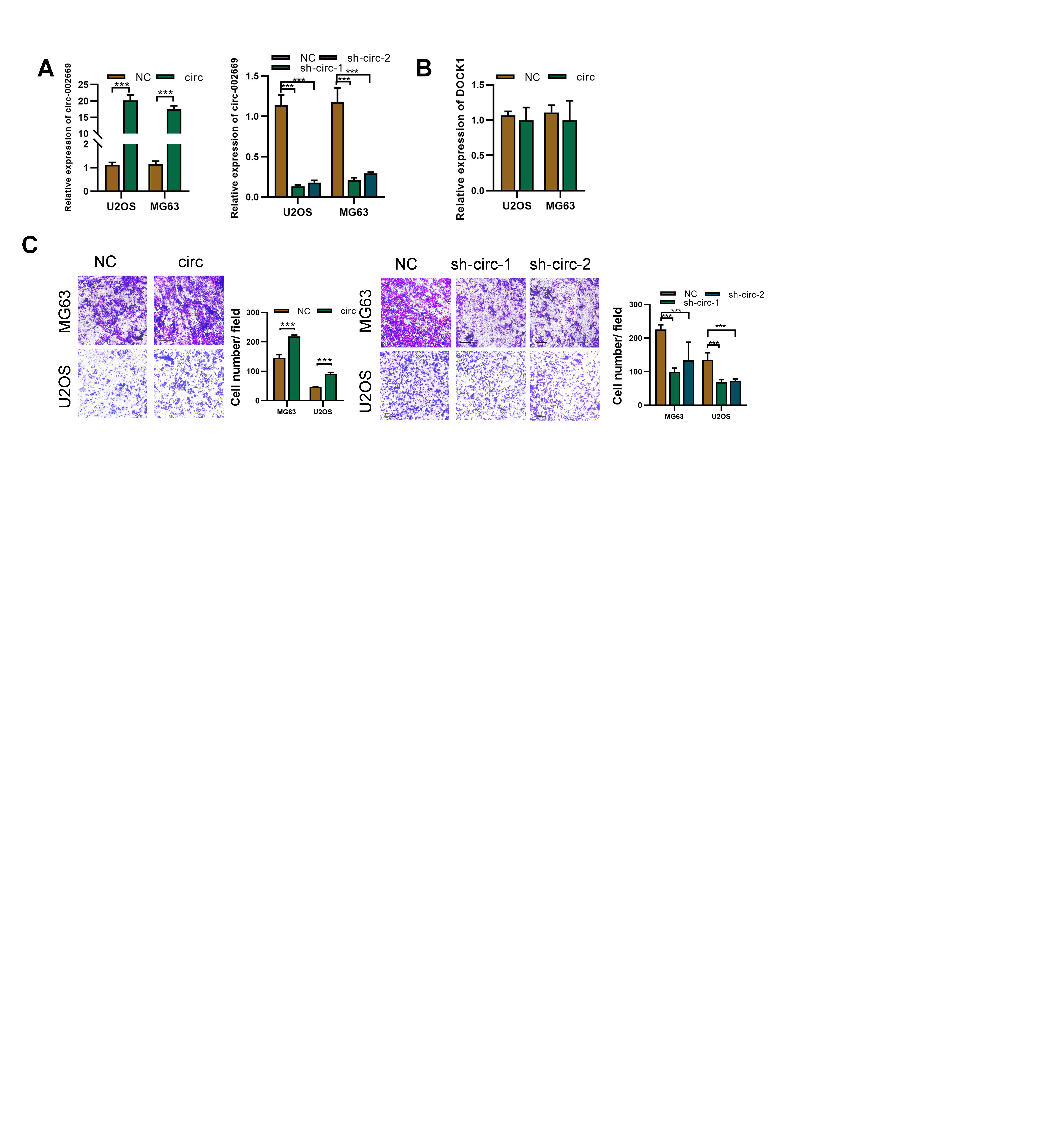

Supplement: Supplementary file 6 — Supplementary Material 6 [file 13062_2024_466_MOESM6_ESM.jpg]

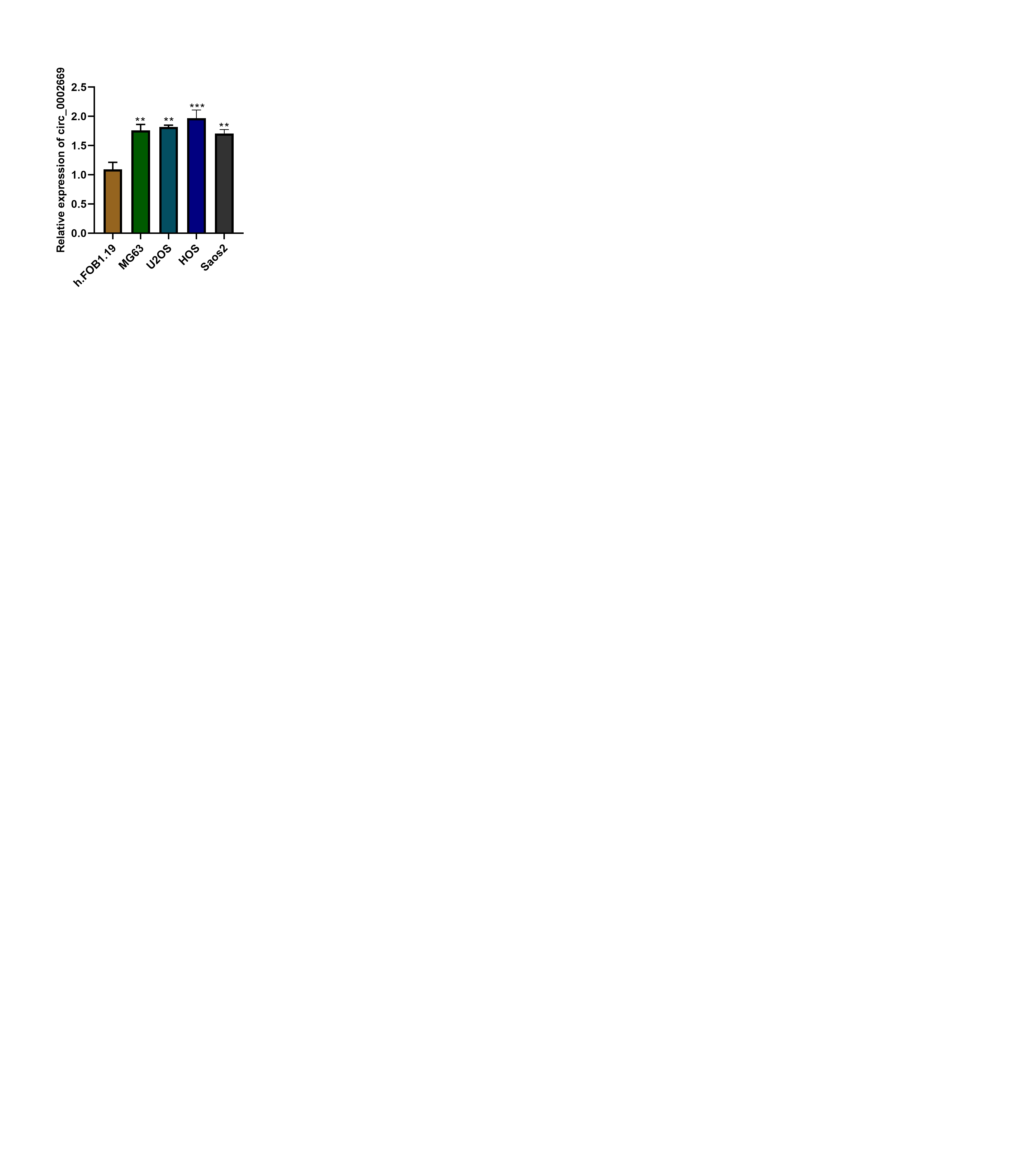

Supplement: Supplementary file 7 — Supplementary Material 7 [file 13062_2024_466_MOESM7_ESM.jpg]
